# The impact of first and second wave of COVID-19 on knee and hip surgeries in Sweden

**DOI:** 10.1186/s40634-021-00382-7

**Published:** 2021-08-13

**Authors:** Andrea Dell’Isola, Ali Kiadaliri, Aleksandra Turkiewicz, Velocity Hughes, Karin Magnusson, Jos Runhaar, Sita Bierma-Zeinstra, Martin Englund

**Affiliations:** 1 Clinical Epidemiology Unit, Orthopedics, Department of Clinical Sciences Lund, Lund University, Wigerthuset, Remissgatan 4, 22185 Lund, Sweden; 2 Centre for Economic Demography, Lund University, Lund, Sweden; 3 Norwegian Institute of Public Health, Cluster for Health Services Research, Oslo, Norway; 4 Department of General Practice, Erasmus MC University Medical Center Rotterdam, Rotterdam, the Netherlands

**Keywords:** COVID-19, Orthopedics, Elective surgeries

## Abstract

**Purpose:**

To investigate the impact of COVID-19 in Sweden on rates of knee and hip surgeries.

**Methods:**

We used healthcare data for the population of the southernmost region in Sweden (1.4 million inhabitants). We did an interrupted time-series analysis to estimate changes in rates and trends of joint replacements (JR), arthroscopies, and fracture surgeries for knee or hip in April–December 2020 compared to pre-COVID-19 levels adjusting for seasonal variations.

**Results:**

We found a drop of 54% (95% CI 42%; 68%) and 42% (95% CI 32%; 52%), respectively, in the rate of JRs and arthroscopies in April 2020 when compared to the counterfactual scenario. This was followed by an increase that brought the rates of JRs and arthroscopies back to their predicted levels also during the beginning of the second wave (November–December 2020). Acute fracture surgeries were largely unaffected, i.e. did not show any decrease as observed for the other surgeries.

**Conclusions:**

In southern Sweden, we observed a marked decrease in elective knee and hip surgeries following the first wave of Covid-19. The rates remained close to normal during the beginning of the second wave suggesting that important elective surgeries for patients with end-stage osteoarthritis can still be offered despite an ongoing pandemic provided adequate routines and hospital resources.

**Supplementary Information:**

The online version contains supplementary material available at 10.1186/s40634-021-00382-7.

## Introduction

The coronavirus disease 2019 (COVID-19) emerged in late 2019 in China causing over a million deaths by the end of the Summer of 2020, and nearly a million more deaths by the end of 2020 when the “second wave” was spreading through the world [8].

In March 2020, many countries imposed lockdowns, in anticipation of the COVID-19 “first wave” and the massive healthcare resources required to meet medical needs, with many hospitals severely curtailing the performance of elective surgeries [7, 16]. Sweden adopted a different strategy, opting for non-binding recommendations discouraging in-person social interactions to reduce the spread of the virus while trying to minimise the unwanted effects caused by lockdowns [9, 15].

Surgeries to treat or alleviate symptoms in RMDs include both elective (e.g. joint replacements (JRs)) as well as emergency surgical procedures, typically for acute fractures. Surgical treatments are often the last resort to alleviate chronic pain, improve function and (or) prevent serious complications [1, 10, 11]. Postponing these surgeries may thus have unwanted consequences on both patients’ and societal perspectives [5, 6, 10]. However, no study has yet reported the effect of the first and second wave of COVID-19 on these surgeries, and in particular not in a country where no formal lockdown was implemented despite a relatively high rate of infection.

Thus, our aim was to investigate the impact of COVID-19 and the Swedish government’s response on the rate of JR, arthroscopies, and surgeries due to fractures in the knee or the hip during the first and second wave of COVID-19.

## Methods

We used register data from Skåne healthcare register for the entire population of Skåne, the southernmost region in Sweden with about 1.4 million inhabitants (13% of the total Swedish population). We included all residents aged ≥18 years anytime during the years 2015 to 2020. Among those, we identified the following surgical procedures of the knee or hip: JR (JR due to fracture excluded), arthroscopy, and surgery due to fracture (including JR) using a combination of the Swedish version of NOMESCO Classification of Surgical Procedures and ICD10 (the International Classification of Diseases, 10th revision) codes (Supplementary files 1A, 1B).

**Fig. 1:**
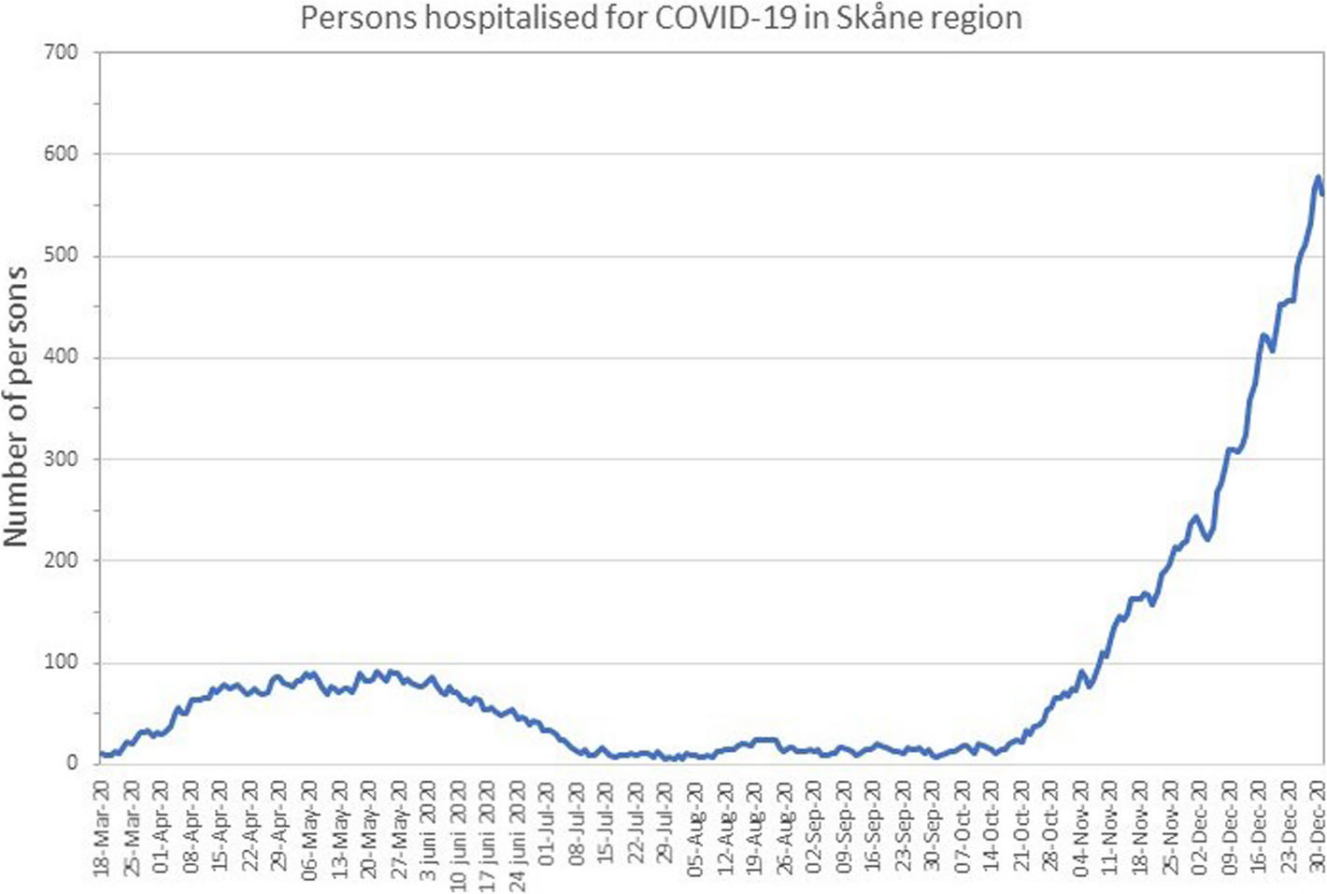
Number of persons hospitalised for COVID-19 per week in the Skåne region. Official Skåne region data retrieved on 19/03/2021 from: https://www.skane.se/digitala-rapporter/lagesbild-covid-19-i-skane/om-statistiken/

To demarcate pre- and post-event periods for the first wave of Covid-19, we established a differentiation point corresponding to mid-March 2020, the time when the Swedish Public Health Agency began recommending social distancing, working from home, distance learning for secondary schools and universities and when the World Health Organisation declared COVID-19 a pandemic [20]. A more detailed timeline of government recommendations is provided in Table 1. Our observation period included also the beginning of the second wave in November–December 2020 when hospital admissions due to Covid-19 in Skåne region reached higher levels than observed in April 2020 (Fig. 1) [19].

**Table 1:** Timeline of the COVID-19 pandemic in Sweden

Date	Event
**2020 - January - 31**	First case of COVID-19 in Sweden confirmed^a^.
**2020 - February - 27**	Five new cases identified^b^.
**2020 - March - 10**	First three cases of COVID-19 reported in the Skåne region^c^.
	The Public Health Agency advised everyone with respiratory infections, even mild cases, to refrain from social contacts. They also ask health care staff working with risk groups to not work if they have any symptoms of respiratory infection. Relatives of elderly should also avoid unnecessary visits at hospitals and in facilities for elderly, and never visit if there are any respiratory symptoms^d^.
**2020 – March - 11**	The Swedish government issued a law limiting freedom of assembly by banning all gatherings larger than 500 people^e^.
**2020 – March - 16**	The Public Health Agency recommended that people over 70 should limit close contact with other people, and avoid crowded areas such as stores, public transport and public spaces^f^.
	The Public Health Agency recommended to work from home^f^.
**2020 – March - 18**	The Swedish government recommend secondary schools and universities to use distance learning^g^.
**2020 – March - 29**	Further restrictions on public gatherings, with limit lowered to 50 people^h^.
**2020 – May – 16**	People over 70 were encouraged to go outside for walks while still following the reccomendations released in March^i^.
**2020 – June – 15**	Reccomendetions on distance learning are lifted^j^
**2020 – November - 24**	Public gatherings and events limited to 8 people, with the exeptions of religious gatherings linked to the death of a person^k^.

^a^: https://www.thelocal.se/20200131/first-case-of-coronavirus-confirmed-in-jonkoping-sweden/?amp

^b^: https://www.folkhalsomyndigheten.se/nyheter-och-press/nyhetsarkiv/2020/februari/ytterligare-fall-av-covid-19-i-flera-regioner/

^c^: https://www.mynewsdesk.com/se/region_skane/pressreleases/pressbulletin-om-covid-19-10-mars-2980402

^d^: https://www.folkhalsomyndigheten.se/nyheter-och-press/nyhetsarkiv/2020/mars/flera-tecken-pa-samhallsspridning-av-covid-19-i-sverige/

^e^: https://www.svt.se/nyheter/snabbkollen/regeringen-stoppar-stora-moten

^f^: https://www.folkhalsomyndigheten.se/nyheter-och-press/nyhetsarkiv/2020/mars/personer-over-70-bor-begransa-sociala-kontakter-tills-vidare/

^g^: https://www.svd.se/presstraff-i-dag-med-utbildningsministern

^h^: https://www.krisinformation.se/nyheter/2020/mars/ytterligare-begransning-sammankomster

^i^: https://www.dn.se/nyheter/sverige/tegnell-rad-om-isolering-till-70-aringar-kan-lattas-i-veckan/

^j^: https://www.svt.se/nyheter/inrikes/lofven-skolorna-oppnar-till-hosten

^k^: https://www.government.se/press-releases/2020/11/maximum-of-eight-people-permitted-at-public-gatherings-and-events/

### Intervention and outcomes

The rate of surgeries per 10,000 adults from March 1st 2015 until December 31st 2020 was used as outcome and grouped into: (i) elective primary JRs which includes all the JR performed every month which were not due to fractures, (ii) emergency knee and hip surgeries due to fractures including JR and other surgical procedures to reduce or fixate the fracture, (iii) elective arthroscopic surgeries such as meniscectomy, synovectomy, exploratory arthroscopies. If more than one surgery (e.g. meniscectomy and ligament reconstruction) was performed at the same time, the procedures were counted separately.

### Statistical analysis

We did an interrupted time-series (ITSA) analysis using segmented ordinary least-squares regression to estimate changes in the levels and trends of surgical procedures compared to pre-COVID-19 levels, adjusting for seasonal variations (Supplementary file 2) [2]. The month of March was treated as a “phase-in” period to give time for the new recommendations to be implemented. In addition, we estimated the absolute and relative difference (with its 95% confidence interval [CI]) between the predicted and the counterfactual scenario in the monthly number of surgeries, where the counterfactual is the expected rate of surgery if COVID-19 had not happened [22]. To account for the possibility that other co-occurring events may be responsible for the observed changes, we assessed changes in the number of surgeries due to fractures, which are normally treated as emergencies that cannot be cancelled or rescheduled. For the surgeries with a sufficient number of cases, we repeated the analysis to study separately people aged 70 years or older who were targeted by specific recommendations advising to avoid any indispensable social interaction.

## Results

### General population

**Fig. 2:**
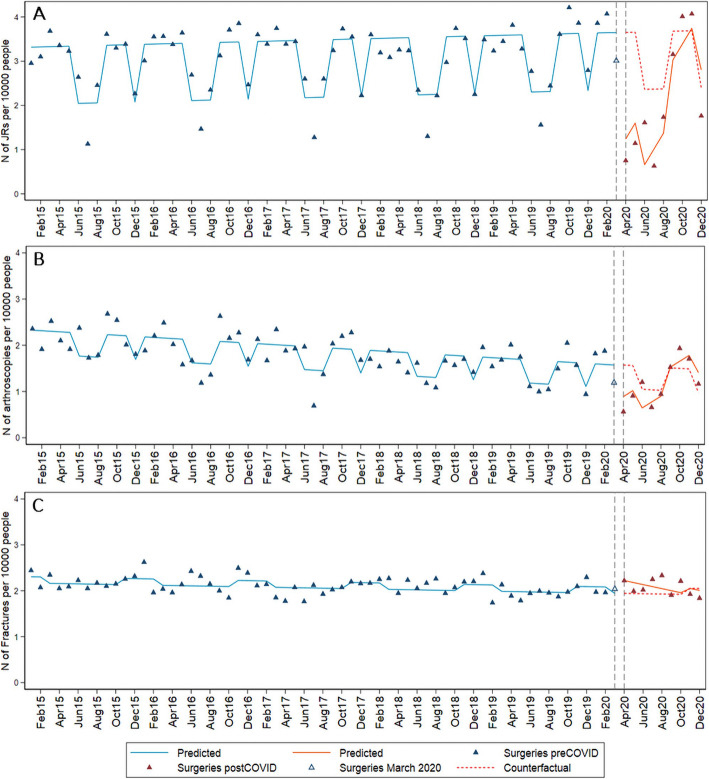
Monthly incidence of surgeries per 10,000 Skåne inhabitants aged > 18. **a** Interrupted time series for JR, **c** arthroscopies, and **d** fracture surgeries

We identified a total of 22,084 JRs, 12,806 arthroscopies and 15,774 fracture surgeries of the knee or hip over the study period. The monthly rate of surgeries and ITSAs are presented in Fig. 2 (supplementary file 3). The results suggest that in April 2020 there was a decrease of 2.41 (95% CI 1.83; 2.98) JRs per 10,000 adults which corresponds to a relative reduction of 66% (95% CI 50%; 81%) when compared to the counterfactual scenario. This was followed by a positive trend signifying a monthly increase of 0.36 (95% CI 0.26; 0.46) JRs per 10,000 adults which brought the rate of JRs to the rate predicted by the counterfactual scenario by October 2020.

The rate of arthroscopies followed a similar pattern with a decrease of 0.68 (95% CI 0.46; 0.90) arthroscopies per 10,000 adults in April, which corresponds to a 43% relative decrease (95% CI 29%; 57%) followed by a positive trend signifying a monthly increase of 0.13 (95% CI: 0.09; 0.17) arthroscopies per 10,000 adults which brought the rate of arthroscopic surgery performed to the rate predicted by the counterfactual scenario by August 2020. The rate of fractures was largely unaffected, with a small increase of 0.28 (95% CI 0.14; 0.41) in April 2020. The reason for surgeries (i.e. main diagnostic code) had a similar distribution in the years pre-COVID and in 2020 (supplementary file 4).

### People aged 70 and over

**Fig. 3:**
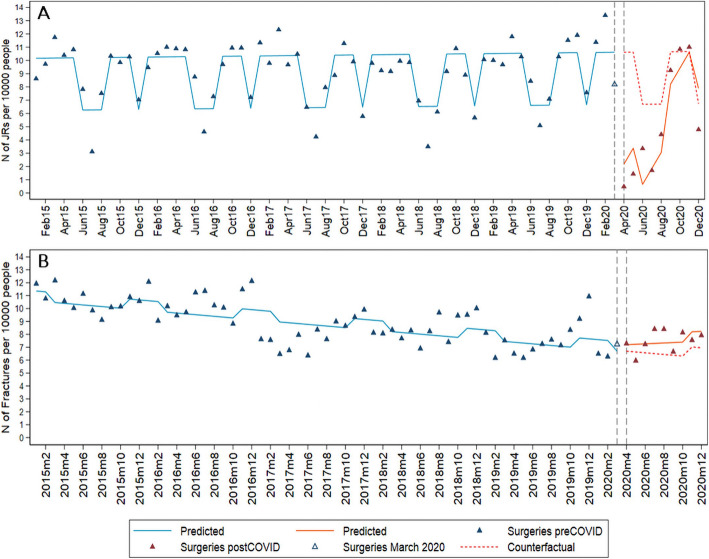
Monthly incidence of surgeries per 10,000 Skåne inhabitants aged ≥70. **a** Interrupted time series for JR, **c** arthroscopies, and **d** fracture surgeries

We identified a total of 3425 JRs, 77 arthroscopies and 3191 fracture surgeries of the knee or hip among people aged 70 or older over the study period (Fig. 3). The results suggest a decrease of 8.44 (95% CI 6.23; 10.65) JRs per 10,000 older adults in April 2020, which corresponds to a relative decrease of 80% (95% CI 59%; 100%) when compared to the counterfactual scenario. This was followed by a positive trend of 1.21 (95% CI 0.85; 1.57) JRs per 10,000 older adults which brought the rate of JRs back to the level predicted by the counterfactual scenario by October 2020. The rate of fracture surgery showed no decrease among older adults in April 2020 with no following trend detected (0.03; 95% CI: − 0.06; 0.13). The number of fractures surgeries appeared to be slightly higher than predicted by the counterfactual scenario from June to December 2020.

## Discussion

In southern Sweden, the first wave of COVID-19 and the government’s recommendation of social distancing were associated with a steep and sudden reduction in the rate of elective knee and hip surgeries among adults, with a more marked reduction among people aged 70 or older. At the same time, no similar reduction was observed for surgeries due to knee or hip fractures.

The observed decrease in elective surgeries is in line with previous reports and was partially expected due to the important disruption triggered by the epidemic [3, 4, 6, 13, 17, 18]. However, none of the available studies implemented a counterfactual model taking into account seasonality and historical trends of surgical procedures. It is known that elective surgeries are strongly affected by seasonality, with lower volumes observable during months that include festivities (e.g. Christmas, summer holidays). In addition, certain procedures like knee arthroscopies have been steadily declining in frequency during the last decade. Studies that did not take into account seasonality and historical trends of surgeries may, therefore, have over- or underestimated the impact of the pandemic.

In southern Sweden, we observed a very sharp decrease in elective surgeries shortly after the Swedish Public Health Agency began recommending social distancing and working from home while the regional health care system advised cancelling all the unnecessary elective surgeries. The cancellation of elective surgery can be interpreted as an attempt to protect patients from nosocomial infection and to preserve resources and personal protective equipment needed in the acute care of COVID-19 patients [6]. Our hypothesis is supported by the relatively low number of COVID-19 infections in the region (Fig. 1). Another possible contributor to the observed decline may be patients cancelling the scheduled surgery due to fear of contracting a COVID-19 infection.

Following the reduction of COVID-19 cases during the summer, the regional health care system advised resuming the elective surgeries with the highest priority which explains the rapid increase in the rate of surgeries observed in August. Interestingly, the rate of JRs and arthroscopies in southern Sweden was in line with the expected rate during the fall of 2020, when the second wave of COVID-19 was spreading. In the Skåne region, the total number of COVID-19 cases and hospitalisation due to COVID-19 were nearly 6 times higher at the beginning of the second wave than during the first wave [6, 13]. The drop in the rate of surgeries in the spring but not in the late fall of 2020 may reflect the initial difficulties the regional health care faced during the first wave, where lack of resources and inadequate organisation may have played an important role. However, caution is needed in the interpretation of the effect of the COVID-19 s wave due to the limited time points available (November–December). The rate of JRs in older adults saw a larger drop compared to the general adult population. This was expected considering that the dedicated recommendations advising people aged 70 or older to avoid any unnecessary social interaction. Overall, we did not observe any essential change in the distribution of the diagnoses (e.g. OA, other rheumatic diseases) for which the surgeries were provided between the period before and after COVID-19. This suggests that no diagnosis-based prioritisation was implemented.

A previous study analysing the rate of JR at the Orthopedic Hospital in Vienna (Austria) during the first and second wave reported similar results showing a rapid decrease in the rate of surgeries in March–April 2020 followed by a recovery that brought the level of surgeries to the expected levels by the fall of 2020 [18]. The authors suggest that the paucity of resources such as personal protective equipment during the first wave and the attempt to spare resources for the care of COVID patients led to the reduction in JRs early on in the pandemic while better preparation during the second wave helped to minimise the disruptions. Another report from a single orthopaedic hospital in Milan (Itlay) shows a larger decrease of hospital admissions and elective surgeries during the second wave than what we observed in southern Sweden [21]. This decrease may be explained by the strict lockdown implemented and the significantly higher rate of COVID infections observed in Italy from September to December 2020.

A similar drop in the rate of elective surgeries for the first wave was recently reported in Norway where an official lockdown was enforced early in the pandemic [13]. Thus, it appears that both lockdowns and non-binding recommendations had a similar impact on the rate of elective surgeries in the initial phase. This suggests that other factors, such as emergency preparation by hospitals, relocation of intensive care staff, and the fear of nosocomial COVID infection spread have played a key role in the cancellation of these surgeries.

Our results indicate no relevant change in the rate of emergency surgeries due to fracture. Despite this result may be expected due to the impossibility to postpone emergency surgeries, numerous reports from countries that implemented strict lockdowns suggest a reduction in the number of emergency surgeries due to fractures [12–14]. Possibly, the non-binding recommendations issued in Sweden may have impacted to a lesser extent on leisure and other activities which contribute to fractures. On the other hand, the pattern of fractures may have changed with a reduction in the number of sport-and leisure-related fractures and an increase in the number of fractures due to domestic accidents as shown in a recent systematic review [12].

Some limitations need to be acknowledged. Due to the limited data available through the register, we were not able to determine if the surgical procedure were postponed by the health care providers or were cancelled by the patient. In addition, we were not able to verify the reason for the fractures which limits our ability to identify a causal factor behind the observed pattern.

## Conclusions

In southern Sweden, the first wave of the COVID-19 pandemic was associated with a marked and rapid drop in the rate of elective knee and hip surgeries which was more evident among older adults. However, the rates returned to normal over few months and stayed that way during the beginning of the second wave, suggesting that adequate preparations had already been made to meet the expected increase of hospital and intensive care admissions. This observation suggests that adequate preparations for a pandemic have the potential to minimise disruption to orthopaedic elective surgeries, often important to RMD patients, can still be carried out.

## Supplementary Information

**Additional file 1: Supplementary file 1A.** Swedish version of NOMESCO Classification of Surgical Procedures and ICD10 (the International Classification of Diseases, 10th revision) codes used to classify hip surgeries. **Supplementary file 1B.** Swedish version of NOMESCO Classification of Surgical Procedures and ICD10 (the International Classification of Diseases, 10th revision) codes used to classify knee surgeries. **Supplementary file 2**. STATA code for main analysis. **Supplementary file 3.** monthly rate of surgery per 10,000 Skåne inhabitants aged > 18. **Supplementary file 4.** Frequency of diagnosis by type of surgery before and during the COVID-19 pandemic.

## Data Availability

Aggregated data used in this study are provided in supplementary file 3.

## Abbreviations

CI: Confidence interval
COVID: Corona virus disease
ITSA: Interrupted time serie
JR: Jpoint replacement
OA: Osteoarthritis
RMD: Rheumatic disease
