# Supplementary material for: The impact of first and second wave of COVID-19 on knee and hip surgeries in Sweden
Source: J Exp Orthop. 2021 Aug 13;8:60. doi: 10.1186/s40634-021-00382-7 (PMC8363236; doi:10.1186/s40634-021-00382-7)
Supplement: Supplementary file 1 — Additional file 1: Supplementary file 1A. Swedish version of NOMESCO Classification of Surgical Procedures and ICD10 (the International Classification of Diseases, 10th revision) codes used to classify hip surgeries. Supplementary file 1B. Swedish version of NOMESCO Classification of Surgical Procedures and ICD10 (the International Classification of Diseases, 10th revision) codes used to classify knee surgeries. Supplementary file 2. STATA code for main analysis. Supplementary file 3. monthly rate of surgery per 10,000 Skåne inhabitants aged > 18. Supplementary file 4. Frequency of diagnosis by type of surgery before and during the COVID-19 pandemic. [file 40634_2021_382_MOESM1_ESM.docx]

**Supplementary file 1A: Swedish version of NOMESCO Classification of Surgical Procedures and ICD10 (the International Classification of Diseases, 10th revision) codes used to classify hip surgeries.**

| **HIP JOINT** | |
| --- | --- |
| **Procedure code (ICD10)** | **Description** |
| **Joint replacement, primary** | |
| **NFB39** | Primary total prosthetic replacement with hybrid technique |
| **NFB49** | Primary total prosthetic replacement with cement |
| **NFB29** | Primary total prosthetic replacement without cement |
| **NFB53** | Primary prosthetic replacement of the patella |
| **NFB59** | Primary prosthetic replacement of the patella with primary total knee replacement |
| **NFB99** | Other primary total prosthetic replacement in the knee |
| **NFB09** | Primary unicompartmental or partial prosthetic replacement without cement |
| **Joint replacement, secondary** | |
| **NFC09** | Secondary half or partial prosthesis in the hip joint without cement |
| **NFC19** | Secondary half or partial prosthesis in the hip joint with cement |
| **NFC20** | Secondary total prosthesis in the hip joint without cement, all parts revised |
| **NFC21** | Secondary total prosthesis in hip joint without cement, cup revision |
| **NFC22** | Secondary total prosthesis in hip joint without cement, stem revision |
| **NFC23** | Secondary total prosthesis in the hip joint without cement, revision of other parts |
| **NFC29** | Secondary total hip replacement without cement, other or unspecified |
| **NFC30** | Secondary total hip replacement with hybrid technology, all parts revised |
| **NFC31** | Secondary total prosthesis in hip joint with hybrid technique, cup audit |
| **NFC32** | Secondary total hip replacement with hybrid technology, stem revision |
| **NFC33** | Secondary total prosthesis in the hip joint with hybrid technology, revision of other parts |
| **NFC39** | Secondary total hip replacement with hybrid technology, other or unspecified |
| **NFC40** | Secondary total prosthesis in the hip joint with cement, all parts revised |
| **NFC41** | Secondary total prosthesis in hip joint with cement, cup revision |
| **NFC42** | Secondary total prosthesis in the hip joint with cement, stem revision |
| **NFC43** | Secondary total prosthesis in the hip joint with cement, revision of other part |
| **NFC49** | Secondary total hip replacement with cement, other or unspecified |
| **NFC59** | Secondary implantation of hip interposition prosthesis |
| **NFC99** | Other secondary joint replacement surgery in the hip joint |
| **Arthroscopies** | |
| **NFE91** | Surgery to the synovial capsule or ligaments |
| **NFF01** | Total synovectomy |
| **NFF11** | Partial synovectomy |
| **NFF21** | Fixation of fragments |
| **NFF31** | Partial excision of articular cartilage |
| **NFF91** | Other operation to the synovial capsule or joint |
| **NFA11** | Exploration of Joint including explorative arthroscopy |
| **NFA01** | Exploration of soft tissues |
| **NFH51** | Excision of intraarticular exostosis or osteophyte |
| **NFH41** | Removal of loose body |
| **NFH91** | Other operation to the knee joint |
| **NFH31** | Adhesions release |
| **Fractures** | |
| **NFJ09** | Closed repositioning of femur fracture |
| **NFJ19** | Open repositioning of femur fracture |
| **NFJ29** | External fixation of femur fracture |
| **NFJ39** | Osteosynthesis of femur fracture with bio-implants |
| **NFJ49** | Osteosynthesis of femur fracture with cerclage, nails, pin or similar |
| **NFJ59** | Osteosynthesis of femur fracture with marrow nail |
| **NFJ69** | Osteosynthesis of femur fracture with plate and screws |
| **NFJ79** | Osteosynthesis of femur fracture with screws only |
| **NFJ89** | Osteosynthesis of femur fracture by other or combined method |
| **NFJ99** | Other surgery for femur fracture |
| **NFB39 (s72)** | Primary total prosthetic replacement with hybrid technique |
| **NFB49 (s72)** | Primary total prosthetic replacement with cement |
| **NFB29 (s72)** | Primary total prosthetic replacement without cement |
| **NFB53 (s72)** | Primary prosthetic replacement of the patella |
| **NFB59 (s72)** | Primary prosthetic replacement of the patella with primary total knee replacement |
| **NFB99 (s72)** | Other primary total prosthetic replacement in the knee |
| **NFB09 (s72)** | Primary unicompartmental or partial prosthetic replacement without cement |

**Supplementary file 1B: Swedish version of NOMESCO Classification of Surgical Procedures and ICD10 (the International Classification of Diseases, 10th revision) codes used to classify knee surgeries.**

| **KNEE JOINT** | |
| --- | --- |
| **Procedure code (ICD10)** | **Description** |
| **Joint replacement, primary** | |
| **NGB39** | Primary total prosthetic replacement with hybrid technique |
| **NGB49** | Primary total prosthetic replacement with cement |
| **NGB29** | Primary total prosthetic replacement without cement |
| **NGB53** | Primary prosthetic replacement of the patella |
| **NGB59** | Primary prosthetic replacement of the patella with primary total knee replacement |
| **NGB99** | Other primary total prosthetic replacement in the knee |
| **NGB09** | Primary unicompartmental or partial prosthetic replacement without cement |
| **NGB19** | Primary unicompartmental or partial prosthetic replacement with cement |
| **Joint replacement, secondary** | |
| **NGC09** | Secondary knee or partial prosthesis without cement |
| **NGC19** | Secondary knee or partial prosthesis with cement |
| **NGC29** | Secondary total knee replacement without cement |
| **NGC39** | Secondary total knee replacement with hybrid technology |
| **NGC49** | Secondary total knee replacement with cement |
| **NGC53** | Secondary patellofemoral prosthesis |
| **NGC59** | Secondary patella prosthesis |
| **NGC99** | Other secondary joint replacement surgery in the knee joint |
| **Arthroscopies** | |
| **NGE01** | Incision or suture of the capsule |
| **NGE11** | Transition or excision of ligaments |
| **NGE41** | Reconstruction of knee ligaments with foreign material |
| **NGE21** | Suture or replantation of knee ligaments |
| **NGE31** | Transposition of knee ligaments |
| **NGE51** | Ligament reconstruction without foreign material |
| **NGE91** | Other operation on knee ligaments |
| **NGF01** | Total synovectomy |
| **NGF11** | Partial synovectomy |
| **NGF21** | Fixation of fragments |
| **NGF31** | Partial excision of articular cartilage |
| **NGF91** | Other operation to the synovial capsule or joint surface |
| **NGD01** | Total excision of the meniscus |
| **NGD11** | Partial excision of the meniscus |
| **NGD21** | Suture and replantation of the knee meniscus |
| **NGD91** | Other operations on the meniscus |
| **NGA01** | Exploration of Joint including explorative arthroscopy |
| **NGA01** | Exploration of soft tissues in the knee or lower leg |
| **NGH51** | Excision of intraarticular exostosis or osteophyte |
| **NGH41** | Removal of loose body |
| **NGH91** | Other operation to the knee joint |
| **NGH31** | Adhesions release |
| **NGA11** | Exploration of knee joint-Arthroscopic or endoscopic |
| **Fractures** | |
| **NGJ09** | Close reduction of fracture in the knee or lower leg |
| **NGJ19** | Open reduction of fracture in the knee or lower leg |
| **NGJ29** | External fixation of fracture in the knee or lower leg |
| **NGJ39** | Osteosynthesis of fracture in the knee or lower leg with bio-implant |
| **NGJ49** | Osteosynthesis of fracture in the knee or lower leg with cerclage, pin or similar |
| **NGJ59** | Osteosynthesis of fracture in the knee or lower leg with marrow nails |
| **NGJ69** | Osteosynthesis of fracture in the knee or lower leg with plates or screws |
| **NGJ79** | Osteosynthesis of fracture in the knee or lower leg with screws only |
| **NGJ89** | Osteosynthesis of fracture in the knee or lower leg with other or combined methods |
| **NGJ99** | Other operation for fractures in the knee or lower leg |
| **NGB39 (S82)** | Primary total prosthetic replacement with hybrid technique after fracture |
| **NGB49 (S82)** | Primary total prosthetic replacement with cement after fracture |
| **NGB29 (S82)** | Primary total prosthetic replacement without cement after fracture |
| **NGB53 (S82)** | Primary prosthetic replacement of the patella after fracture |
| **NGB59 (S82)** | Primary prosthetic replacement of the patella with primary total knee replacement after fracture |
| **NGB99 (S82)** | Other primary total prosthetic replacement in the knee after fracture |
| **NGB09 (S82)** | Primary unicompartmental or partial prosthetic replacement without cement after fracture |
| **NGB19 (S82)** | Primary unicompartmental or partial prosthetic replacement with cement after fracture |

**Supplementary file 2: STATA code for main analysis**

//Joint replacement

itsa JointReplacement season1, single trperiod(2020m3) replace lag (12) posttend figure

// Arthroscopies

itsa Arthroscopies season1, single trperiod(2020m3) replace lag (12) posttend figure

//Fractures

itsa Fractures season2, single trperiod(2020m3) replace lag (12) posttend figure

**Season1** = Dummy variable. June, July, August and December have value 1 to identify holydays.

**Season2**= Dummy variable. November, December, January, February have value 1 to identify months with higher rate of fractures.

**Supplementary file 3: monthly rate of surgery per 10,000 Skåne inhabitants aged > 18**

| Year | Month | Joint replacement | Fractures | Arthroscopies |
| --- | --- | --- | --- | --- |
| 2015 | January | 2.95 | 2.44 | 2.36 |
| 2015 | February | 3.10 | 2.07 | 1.91 |
| 2015 | March | 3.68 | 2.35 | 2.52 |
| 2015 | April | 3.36 | 2.05 | 2.10 |
| 2015 | May | 3.23 | 2.09 | 1.91 |
| 2015 | June | 2.64 | 2.23 | 2.38 |
| 2015 | July | 1.13 | 2.05 | 1.73 |
| 2015 | August | 2.45 | 2.17 | 1.79 |
| 2015 | September | 3.61 | 2.10 | 2.68 |
| 2015 | October | 3.30 | 2.15 | 2.54 |
| 2015 | November | 3.39 | 2.26 | 2.01 |
| 2015 | December | 2.27 | 2.32 | 1.81 |
| 2016 | January | 3.01 | 2.62 | 1.88 |
| 2016 | February | 3.55 | 1.96 | 2.20 |
| 2016 | March | 3.56 | 2.04 | 2.49 |
| 2016 | April | 3.38 | 1.96 | 2.02 |
| 2016 | May | 3.64 | 2.14 | 1.58 |
| 2016 | June | 2.69 | 2.43 | 1.67 |
| 2016 | July | 1.47 | 2.32 | 1.18 |
| 2016 | August | 2.35 | 2.15 | 1.36 |
| 2016 | September | 3.13 | 2.00 | 2.63 |
| 2016 | October | 3.71 | 1.84 | 2.16 |
| 2016 | November | 3.85 | 2.50 | 2.27 |
| 2016 | December | 2.47 | 2.39 | 1.69 |
| 2017 | January | 3.60 | 2.11 | 2.13 |
| 2017 | February | 3.39 | 2.14 | 1.67 |
| 2017 | March | 3.74 | 1.85 | 2.34 |
| 2017 | April | 3.39 | 1.78 | 1.88 |
| 2017 | May | 3.45 | 2.07 | 1.93 |
| 2017 | June | 2.60 | 1.77 | 1.97 |
| 2017 | July | 1.28 | 2.12 | 0.69 |
| 2017 | August | 2.60 | 1.93 | 1.37 |
| 2017 | September | 3.24 | 2.03 | 2.03 |
| 2017 | October | 3.73 | 2.07 | 2.20 |
| 2017 | November | 3.55 | 2.20 | 2.27 |
| 2017 | December | 2.23 | 2.16 | 1.68 |
| 2018 | January | 3.60 | 2.17 | 1.70 |
| 2018 | February | 3.19 | 2.25 | 1.54 |
| 2018 | March | 3.09 | 2.27 | 1.88 |
| 2018 | April | 3.26 | 1.95 | 1.64 |
| 2018 | May | 3.24 | 2.23 | 1.41 |
| 2018 | June | 2.35 | 2.05 | 1.61 |
| 2018 | July | 1.30 | 2.17 | 1.18 |
| 2018 | August | 2.22 | 2.26 | 1.08 |
| 2018 | September | 2.97 | 1.95 | 1.66 |
| 2018 | October | 3.74 | 2.07 | 1.57 |
| 2018 | November | 3.51 | 2.19 | 1.70 |
| 2018 | December | 2.25 | 2.20 | 1.42 |
| 2019 | January | 3.49 | 2.38 | 1.96 |
| 2019 | February | 3.23 | 1.74 | 1.54 |
| 2019 | March | 3.45 | 2.13 | 1.68 |
| 2019 | April | 3.82 | 1.89 | 2.01 |
| 2019 | May | 3.28 | 1.79 | 1.75 |
| 2019 | June | 2.77 | 1.95 | 1.11 |
| 2019 | July | 1.56 | 1.99 | 1.00 |
| 2019 | August | 2.44 | 1.96 | 1.04 |
| 2019 | September | 3.61 | 1.87 | 1.49 |
| 2019 | October | 4.21 | 1.97 | 2.05 |
| 2019 | November | 3.86 | 2.10 | 1.57 |
| 2019 | December | 2.79 | 2.29 | 0.94 |
| 2020 | January | 3.86 | 1.97 | 1.82 |
| 2020 | February | 4.07 | 1.96 | 1.88 |
| 2020 | March | 3.01 | 2.04 | 1.19 |
| 2020 | April | 0.75 | 2.22 | 0.56 |
| 2020 | May | 1.14 | 1.99 | 0.91 |
| 2020 | June | 1.61 | 2.02 | 1.20 |
| 2020 | July | 0.63 | 2.25 | 0.66 |
| 2020 | August | 1.73 | 2.33 | 0.94 |
| 2020 | September | 3.15 | 1.91 | 1.52 |
| 2020 | October | 4.01 | 2.21 | 1.93 |
| 2020 | November | 4.07 | 1.92 | 1.71 |
| 2020 | December | 1.76 | 1.84 | 1.16 |

**Supplementary file 4: Frequency of diagnosis by type of surgery before and during the COVID-19 pandemic.**

| Surgery | Diagnosis^£^ | Before COVID-19 pandemic, n(%)* | During COVID-19 pandemic, n(%)^#^ |
| --- | --- | --- | --- |
| Joint replacements |  |  |  |
|  | OA | 17464 (92) | 2846 (91) |
|  | Other | 1517 (8) | 288 (9) |
| Arthroscopies | Meniscus | 4477 (65) | 712 (68) |
|  | OA | 832 (12) | 100 (10) |
|  | Other | 1622 (23) | 232 (22) |
| Fractures | Hip | 10943 (84) | 2154 (84) |
|  | Lower leg | 1288 (10) | 238 (9) |
|  | Other | 796 (6) | 163 (6) |
| ^£^ Only main diagnostic code reported | | | |
| * 1^st^ January 2005 / 29^th^ February 2020 | | | |
| ^#^1^st^ March 2020 / 31^st^ December 2020 | | | |
